# Supplementary material for: Publishing, signaling, social capital, and gender: Determinants of becoming a tenured professor in German political science
Source: PLoS One. 2021 Jan 6;16(1):e0243514. doi: 10.1371/journal.pone.0243514 (PMC7787375; doi:10.1371/journal.pone.0243514)
Supplement: S1 File — (DOCX) [file pone.0243514.s001.docx]

Online Annex

# Reputable and regular/undecided publishers

## Agreed on high reputation:

`"Amsterdam University Press (AUP)"' `"Barbara Budrich"' `"C. H. Beck"' `"Cambridge University Press"' `"Campus Verlag"' `"Cornell University Press"' `"DVA"' `"Deutsche Verlagsanstalt"' `"Dietz Verlag"' `"Duncker & Humblot"' `"Edition Sigma"' `"Edward Elgar Publishing"' `"Elsevier"' `"Kluwer Academic"' `"Leske + Budrich"' `"MIT Press"' `"Manchester University Press (MUP)"' `"Nomos Verlag"' `"Oxford University Press (OUP)"' `"Palgrave Macmillan"' `"Princeton University Press"' `"Routledge"' `"Rowohlt Verlag"' `"SAGE Publishing"' `"Springer"' `"Springer Gabler"' `"Springer VS (Verlag für Sozialwissenschaften)"' `"Suhrkamp Verlag"' `"Taylor & Francis"' `"University of Chicago Press"' `"University of Minnesota Press"' `"University of Toronto Pres s"' `"W. Bertelsmann Verlag"' `"Westdeutscher Verlag"' `"dtv Verlagsgesellschaft"' `"edition sigma"'

## Agreed on regular reputation or not agreed:

`"AFES-Press"' `"AV Akademikerverlag"' `"Academia"' `"Agenda"' `"Akademie Verlag"' `"Akademische Verlagsgemeinschaft München"' `"Akademischer Verlag"' `"Anchor Academic Publishing"' `"Anna Blume"' `"Argument Verlag"' `"Armand Colin"' `"Arnold-Bergstraesser-Institut"' `"Ashgate Publishing"' `"Ateliers - Henry Douger"' `"Aufbau-Verlag"' `"Avebury"' `"BBJ Consult"' `"BIS-Verlag"' `"Barrister & Principal"' `"Bautz Verlag"' `"Berlin University Press"' `"Berlin-Verlag Spitz"' `"Berliner Debatte Wissenschaftsverlag"' `"Berliner Wissenschafts-Verlag"' `"Bibliotheka Edizioni"' `"Bier'sche Verlagsanstalt"' `"Bildungsverlag EINS"' `"Birkhäuser Basel"' `"Books on Demand (BoD)"' `"Bouvier-Verlag"' `"Brasilienkunde Verlag"' `"Breitenbach"' `"Brill | Nijhof Digital Publishing"' `"De Gruyter"' `"Deutscher Instituts-Verlag"' `"Deutscher Universitäts-Verlag"' `"Dietrich"' `"Diplomica Verlag"' `"Drewipunkt GmbH"' `"Droste Verlag"' `"EDUSC"' `"ENFORCER Pülz"' `"Econ"' `"Economica Verlag"' `"Edinburgh University Press"' `"Edition Paideia"' `"Edition Passagen/Böhlau Verlag"' `"Edition Temmen"' `"Editora Unimonte"' `"Editora Unimontes"' `"Elgar Verlag"' `"Ergon-Verlag"' `"Erich Schmidt Verlag"' `"Europa Union Verlag"' `"European Consortium for Political Research (ECPR)"' `"Europäische Verlagsgesellschaft"' `"FAU University Press"' `"Financial Times Energy Publishing"' `"Fontamara"' `"Franz Steiner Verlag"' `"GIGA Verlag"' `"GRIN Verlag"' `"Gabriele Schäfer Verlag"' `"Gordon+Breach Verlag Fakultas"' `"Gower"' `"Haag und Herchen Verlag"' `"Hamburger Edition"' `"Hampp Verlag"' `"Hart Publishing"' `"Haupt"' `"Haymarket"' `"Herder Verlag"' `"Hugendubel-Verlag"' `"Humanitas Verlag"' `"I.B. Tauris"' `"ISP Verlag"' `"Ibidem-Verlag"' `"Innsbruck University Press"' `"J.B. Metzler"' `"JAI Press"' `"Junius Verlag"' `"K. G. Saur"' `"Kassel University Press (KUP)"' `"Kein Verlag"' `"KiWi-Taschenbuch"' `"Klartext Verlag"' `"Klaus Schwarz Verlag"' `"Klett Perthes Verlag"' `"Klett-Cotta"' `"Klinkhardt"' `"Knowledge World Publishers (KW Publishers)"' `"Kohlhammer Verlag"' `"Konkret Literatur Verlag"' `"Königshausen & Neumann"' `"Kösel-Verlag"' `"LAIKA-Verlag"' `"LZT Verlag"' `"La Tribu Ediciones"' `"Lambertus"' `"Lamuv Verlag"' `"Le Cavalier Bleu"' `"Leiden University Press"' `"Leipziger Universitätsverlag"' `"Lexikus"' `"Lexington Books"' `"Links-Verlag"' `"Lit Verlag"' `"Logos Verlag"' `"Longman Publisher"' `"Louisoder Verlag"' `"Luchterhand"' `"Lukas Verlag"' `"Lynne Rienner Publishers"' `"Löcker Verlag"' `"Madbuli"' `"Mandelbaum Verlag"' `"Mannheim University Press"' `"Mantis Verlag"' `"Marix Verlag"' `"Martin Meidenbauer Verlag."' `"Matthias-Grünewald-Verlag"' `"Metropol-Verlag"' `"Metropolis"' `"Minerva Press"' `"Modern Humanities Research Association (MHRA)"' `"Mohr Siebeck Verlag"' `"Müller + Bass"' `"NWB Verlag"' `"New Academic Press"' `"Nueva Trilce"' `"Oekom-Verlag"' `"Oldenbourg Verlag"' `"Olms"' `"Olzog"' `"Optimus Verlag"' `"Orell Füssli"' `"PLUTO PR"' `"Pahl-Rugenstein"' `"Parerga-Verlag"' `"Passagen Verlag"' `"Paulo Freire Verlag"' `"Paulusverlag"' `"Peter-Lang-Verlagsgruppe"' `"Pfaffenweiler"' `"Philosophie im Elfenbeinturm"' `"Physica-Verlag"' `"Pinter Publishers"' `"Piper Verlag"' `"Potsdam University Press"' `"Pro Universitate Verlag"' `"Prolog"' `"Quorum Verlag"' `"Reclam Verlag"' `"Redline Verlag"' `"Resch-Verlag"' `"Rheinland-Verlag"' `"Richard Boorberg Verlag"' `"Riva"' `"Rosspen"' `"Rotbuch Verlag"' `"Rotpunktverlag"' `"Rowman & Littlefield International-Verlag"' `"Scoventa Verlag"' `"Seismo"' `"Sense Publishers"' `"Service Fachverlag"' `"Siedler"' `"Sigmaringen"' `"Societäts-Verlag"' `"Sonstiges"' `"Sosyal Arastirmalar Vakfi"' `"Stark"' `"Straube Verlag"' `"Synchron"' `"Südwestdeutscher Verlag"' `"TVV Verlag"' `"Tectum Verlag"' `"Tectum Wissenschaftsverlag"' `"Temple University Press"' `"Textem Verlag"' `"Trade Focus Verlag"' `"Truppendienst"' `"Tuduv Studie"' `"UTB"' `"UVK Verlagsgesellschaft"' `"Ullstein"' `"United States Institute of Peace nior & Pressler"' `"Verlag Julius Klinkhardt"' `"Verlag Karl Alber"' `"Verlag Matthes & Seitz Berlin"' `"Verlag Schulz-Kirchner"' `"Verlag Soziale Hilfe"' `"Verlag Vögel"' `"Verlag Westfälisches Dampfboot"' `"Verlag Wissenschaft und Politik"' `"Verlagrts-Verlag"' `"Wagenbach"' `"Wallstein Verlag"' `"Waxmann Verlag"' `"Wehrhahn Verlag"' `"Westfälisches Dampfboot"' `"Wilhelm Fink Verlag"' `"Winkler Verlag"' `"Wissenschaftliche Buchgesellschaft"' `"Wissenschaftlicher Verlag Berlin"' `"Wissenschaftlicher Verlag Trier"' `"Wissenschaftsverlag Rothe"' `"Wochenschau Verlag"' `"World Scientific Publishing"' `"XS-Verlag"' `"ZEI Verlag"' `"Zed Books"' `"ars una"' `"b_books"' `"edition fatal"' `"edition-tranvia"' `"epodium"' `"l’Harmattan"' `"res publica Wissenschaftsverlag"' `"transcript Verlag"' `"transfer verlag"' `"utzverlag"' `"Éditions Klincksieck"' `"ça-ira-Verlag"'

# Descriptive data

Table A1: Descriptive data for all researchers

| Variable | Mean | Std. Dev. | Min | Max |
| --- | --- | --- | --- | --- |
|  |  |  |  |  |
| Female | 0.38 | 0.49 | 0 | 1 |
| SSCI journal articles | 1.73 | 2.89 | 0 | 26.67 |
| Non-SSCI journal articles | 3.37 | 5.4 | 0 | 79.47 |
| Monographs, reputable | 0.61 | 0.98 | 0 | 18 |
| Monographs, regular | 0.61 | 1.35 | 0 | 28 |
| Edited volumes | 0.85 | 1.66 | 0 | 13.57 |
| Book chapters | 7.09 | 10.39 | 0 | 88.33 |
| Gray literature | 6.18 | 12.76 | 0 | 242 |
| Years since habilitation | 1.04 | 3.06 | 0 | 30 |
| Years since junior prof | 0.48 | 1.73 | 0 | 15 |
| University of excellence | 0.29 | 0.39 | 0 | 1 |
| Months abroad | 17.04 | 28.81 | 0 | 235 |
| Graduated abroad | 0.16 | 0.37 | 0 | 1 |
| PhD abroad | 0.11 | 0.32 | 0 | 1 |
| International publications | 7.1 | 9.9 | 0 | 69 |
| Awards | 0.26 | 0.71 | 0 | 7 |
| DFG funding | 0.17 | 0.54 | 0 | 6 |
| Mobility | 1.92 | 1.82 | 0 | 10 |
| Interim professor | 0.45 | 0.99 | 0 | 10 |
| Co-authors | 14.43 | 23.03 | 0 | 294 |
| Incomplete | 0.13 | 0.33 | 0 | 1 |
| Childless | 0.32 | 0.47 | 0 | 1 |
| Parent | 0.32 | 0.47 | 0 | 1 |
| No child info | 0.36 | 0.48 | 0 | 1 |

Data from 1,453 individuals and 36,875 observations.

Our dataset contains 1453 individuals, among which 38 percent are female. An average researcher in our dataset has 1.73 SSCI publications, 3.37 non-SSCI articles, 0.61 monographs from reputable publishers and 0.61 monographs from other publishing houses, 0.85 edited volumes, and about 6 pieces of gray literature (all co-author adjusted). Only a few researchers have got a habilitation or a junior professorship, so the average time spent after each are only 1.04 resp. 0.48 years. 29 percent of all career steps in our dataset took place in a university that held the status “university of excellence” at least once. Researchers spent an average of 17 months abroad, 16 percent graduated abroad, and 11 percent received their PhD from abroad. Researchers have an average of 7 non-German publications, received 0.26 awards, and 17 percent received DFG funding once or more. They have changed place almost twice, and acted as an interim-professor 0.45 times. They have 14.4 co-authors, and publication lists are incomplete for 13 percent. Finally, 32 percent are childless at the time of the survey, 32 percent have at least one child, and data on children is missing for the remaining 36 percent.

# Additional regressions

Table A2: Replication with non-logged values

|  | (4) | (5) | (6) | (7) |
| --- | --- | --- | --- | --- |
|  | Social capital | Children | Women | Men |
| Female | 1.23 |  |  |  |
|  | (1.52) |  |  |  |
| SSCI journal articles | 1.09^***^ | 1.09^***^ | 1.12^*^ | 1.09^***^ |
|  | (4.37) | (4.19) | (2.31) | (4.02) |
| Monographs, reputable | 1.05 | 1.04 | 1.10 | 1.04 |
|  | (0.81) | (0.73) | (0.66) | (0.49) |
| Monographs, regular | 0.92 | 0.93 | 1.15 | 0.88^*^ |
|  | (-1.46) | (-1.40) | (0.82) | (-2.12) |
| Non-SSCI journal articles | 1.01 | 1.01 | 1.03 | 1.01 |
|  | (1.03) | (0.81) | (0.85) | (1.12) |
| Edited volumes | 1.05 | 1.05 | 0.90 | 1.08 |
|  | (1.06) | (1.09) | (-0.90) | (1.61) |
| Book chapters | 1.03^***^ | 1.03^***^ | 1.04^+^ | 1.02^**^ |
|  | (3.43) | (3.35) | (1.87) | (2.85) |
| Gray literature | 1.00 | 1.00 | 1.02 | 1.00 |
|  | (0.62) | (0.57) | (0.93) | (0.45) |
| Years since habilitation | 1.66^***^ | 1.66^***^ | 1.55^**^ | 1.82^***^ |
|  | (7.55) | (7.52) | (3.11) | (7.65) |
| Years since habilitation² | 0.96^***^ | 0.96^***^ | 0.96^*^ | 0.96^***^ |
|  | (-5.20) | (-5.18) | (-2.57) | (-5.03) |
| Years since ass prof | 1.50^***^ | 1.50^***^ | 0.97 | 1.84^***^ |
|  | (5.39) | (5.53) | (-0.17) | (7.46) |
| Years since junior prof² | 0.97^**^ | 0.97^***^ | 1.01 | 0.95^***^ |
|  | (-3.25) | (-3.38) | (0.57) | (-4.99) |
| International publications | 1.01 | 1.01 | 1.01 | 1.01 |
|  | (1.22) | (1.20) | (0.27) | (1.18) |
| Months abroad | 1.00 | 1.00 | 1.00 | 1.00 |
|  | (1.20) | (1.30) | (0.87) | (1.06) |
| Graduated abroad | 1.04 | 1.06 | 1.07 | 1.03 |
|  | (0.18) | (0.27) | (0.16) | (0.14) |
| PhD abroad | 1.43 | 1.43 | 1.13 | 1.90^*^ |
|  | (1.60) | (1.63) | (0.28) | (2.35) |
| University of excellence | 0.64^**^ | 0.62^**^ | 0.60^+^ | 0.66^*^ |
|  | (-2.75) | (-2.91) | (-1.71) | (-2.05) |
| Awards | 1.41^***^ | 1.42^***^ | 1.59^***^ | 1.37^***^ |
|  | (5.11) | (5.19) | (3.49) | (4.09) |
| DFG funding | 1.26^*^ | 1.28^**^ | 1.42 | 1.31^**^ |
|  | (2.47) | (2.77) | (1.30) | (2.97) |
| Mobility | 1.27^***^ | 1.27^***^ | 1.26^***^ | 1.29^***^ |
|  | (6.97) | (7.09) | (3.76) | (5.93) |
| Interim professor | 0.99 | 0.99 | 1.17 | 0.93 |
|  | (-0.22) | (-0.12) | (1.40) | (-0.83) |
| Co-authors | 1.00 | 1.00 | 1.00 | 1.00 |
|  | (0.89) | (0.92) | (-0.59) | (1.25) |
| Childless man |  | 1.00 |  | 1.00 |
|  |  | (.) |  | (.) |
| Childless Woman |  | 1.16 | 1.00 |  |
|  |  | (0.59) | (.) |  |
| Father |  | 1.20 |  | 1.24 |
|  |  | (1.01) |  | (1.17) |
| Mother |  | 1.21 | 1.08 |  |
|  |  | (0.74) | (0.27) |  |
| W/o child info man |  | 0.95 |  | 0.97 |
|  |  | (-0.25) |  | (-0.18) |
| W/o child info woman |  | 1.54^+^ | 1.16 |  |
|  |  | (1.91) | (0.48) |  |
| Before 2002 | 1.44^*^ | 1.46^**^ | 1.10 | 1.68^**^ |
|  | (2.50) | (2.67) | (0.27) | (3.20) |
| Incomplete | 1.96^***^ | 1.93^***^ | 2.21^*^ | 1.95^***^ |
|  | (4.02) | (3.89) | (2.12) | (3.30) |
| r2 | .12 | .12 | .14 | .15 |
| Individuals tenured | 356 | 356 | 109 | 247 |
| Individuals total | 1453 | 1453 | 550 | 903 |
| Observations | 35578 | 35578 | 10203 | 25375 |

Notes: Exponentiated coefficients; t statistics in parentheses; cluster-robust standard errors;

^+^ *p* < 0.1, ^*^ *p* < 0.05, ^**^ *p* < 0.01, ^***^ *p* < 0.001

Table A3: Different effects on women vs men

|  | (1) |  |
| --- | --- | --- |
|  | Men women  interaction |  |
| Female=1 | 0.75 | (-0.59) |
| SSCI journal articles (ln) | 1.62^***^ | (4.13) |
| Female=1 # SSCI journal articles (ln) | 1.15 | (0.58) |
| Monographs, reputable (ln) | 1.02 | (0.11) |
| Female=1 # Monographs, reputable (ln) | 1.03 | (0.08) |
| Monographs, regular (ln) | 0.72^*^ | (-2.19) |
| Female=1 # Monographs, regular (ln) | 1.99^*^ | (1.96) |
| Non-SSCI journal articles (ln) | 1.13 | (1.12) |
| Female=1 # Non-SSCI journal articles (ln) | 0.95 | (-0.26) |
| Edited volumes (ln) | 1.33^+^ | (1.80) |
| Female=1 # Edited volumes (ln) | 0.58 | (-1.54) |
| Book chapters (ln) | 1.19 | (1.39) |
| Female=1 # Book chapters (ln) | 1.77^*^ | (2.17) |
| Gray literature (ln) | 1.04 | (0.46) |
| Female=1 # Gray literature (ln) | 1.08 | (0.46) |
| Years since habilitation | 1.69^***^ | (6.74) |
| Female=1 # Years since habilitation | 0.88 | (-0.77) |
| Years since habilitation² | 0.96^***^ | (-4.67) |
| Female=1 # Years since habilitation² | 1.00 | (0.14) |
| Years since ass prof | 1.78^***^ | (6.73) |
| Female=1 # Years since ass prof | 0.59^**^ | (-2.69) |
| Years since junior prof² | 0.96^***^ | (-4.35) |
| Female=1 # Years since junior prof² | 1.05^*^ | (2.45) |
| International publications (ln) | 1.06 | (0.52) |
| Female=1 # International publications (ln) | 0.88 | (-0.59) |
| Months abroad (ln) | 1.09 | (1.56) |
| Female=1 # Months abroad (ln) | 1.01 | (0.06) |
| Graduated abroad | 1.01 | (0.03) |
| Female=1 # Graduated abroad | 1.10 | (0.20) |
| PhD abroad | 2.09^**^ | (3.22) |
| Female=1 # PhD abroad | 0.61 | (-1.01) |
| University of excellence | 0.55^**^ | (-2.94) |
| Female=1 # University of excellence | 0.99 | (-0.02) |
| Awards (ln) | 1.77^**^ | (3.08) |
| Female=1 # Awards (ln) | 1.26 | (0.69) |
| DFG funding (ln) | 1.76^***^ | (3.43) |
| Female=1 # DFG funding (ln) | 0.92 | (-0.19) |
| Mobility (ln) | 2.46^***^ | (6.09) |
| Female=1 # Mobility (ln) | 1.00 | (-0.02) |
| Interim professor (ln) | 1.03 | (0.20) |
| Female=1 # Interim professor (ln) | 1.49 | (1.31) |
| Co-authors (ln) | 1.11 | (1.10) |
| Female=1 # Co-authors (ln) | 0.89 | (-0.70) |
| with children | 1.34 | (1.60) |
| w/o child info | 0.98 | (-0.10) |
| Female=1 # with children | 0.82 | (-0.60) |
| Female=1 # w/o child info | 1.24 | (0.59) |
| Before 2002 | 1.63^**^ | (2.83) |
| Female=1 # Before 2002 | 0.71 | (-0.90) |
| Incomplete | 2.34^***^ | (3.80) |
| Female=1 # Incomplete | 1.03 | (0.06) |
| r2 | .15 |  |
| Individuals tenured | 356 |  |
| Individuals total | 1453 |  |
| Observations | 35578 |  |

Notes: Exponentiated coefficients; t statistics in parentheses; cluster-robust standard errors;

^+^ *p* < 0.1, ^*^ *p* < 0.05, ^**^ *p* < 0.01, ^***^ *p* < 0.001

Figure A1: Visualization of how effect on women are different than on men

Table A4: Robustness tests

|  | (1) | (2) | (3) | (4) | (5) | (6) |
| --- | --- | --- | --- | --- | --- | --- |
|  | Impact  factor | Incomplete  dropped | Post  2002 | Only  professors | Univ of  excellence | Nr  children |
| Female | 1.17 | 1.25 | 1.15 | 1.11 | 1.23 |  |
|  | (1.06) | (1.45) | (0.95) | (0.77) | (1.41) |  |
| SSCI journal articles (ln) # impact | 1.66^***^ |  |  |  |  |  |
|  | (4.26) |  |  |  |  |  |
| SSCI journal articles (ln) |  | 1.77^***^ | 1.79^***^ | 1.22^*^ | 1.81^***^ | 1.65^***^ |
|  |  | (5.25) | (5.20) | (2.05) | (5.07) | (5.01) |
| Monographs, reputable (ln) | 1.13 | 1.10 | 1.19 | 0.87 | 1.07 | 1.02 |
|  | (0.84) | (0.66) | (1.19) | (-1.07) | (0.45) | (0.17) |
| Monographs, regular (ln) | 1.01 | 1.02 | 0.83 | 0.95 | 0.96 | 0.90 |
|  | (0.10) | (0.12) | (-1.25) | (-0.36) | (-0.29) | (-0.83) |
| Non-SSCI journal articles (ln) | 1.17 | 1.12 | 1.06 | 1.05 | 1.04 | 1.09 |
|  | (1.59) | (1.10) | (0.55) | (0.55) | (0.38) | (0.94) |
| Edited volumes (ln) | 1.14 | 1.12 | 1.38^*^ | 0.91 | 1.32^+^ | 1.22 |
|  | (0.91) | (0.80) | (2.25) | (-0.82) | (1.92) | (1.47) |
| Book chapters (ln) | 1.54^***^ | 1.46^**^ | 1.30^*^ | 1.15 | 1.22^+^ | 1.36^**^ |
|  | (3.35) | (2.93) | (2.05) | (1.33) | (1.66) | (2.81) |
| Gray literature (ln) | 1.07 | 1.08 | 1.12 | 1.03 | 1.07 | 1.05 |
|  | (0.87) | (0.96) | (1.46) | (0.48) | (0.92) | (0.69) |
| Years since habilitation | 1.68^***^ | 1.67^***^ | 1.38^***^ | 1.36^***^ | 1.60^***^ | 1.57^***^ |
|  | (5.96) | (5.92) | (4.78) | (4.37) | (5.97) | (6.72) |
| Years since habilitation² | 0.95^***^ | 0.95^***^ | 0.97^***^ | 0.98^*^ | 0.96^***^ | 0.97^***^ |
|  | (-4.77) | (-4.65) | (-3.87) | (-2.15) | (-4.20) | (-4.72) |
| Years since ass prof | 1.33^***^ | 1.38^***^ | 1.45^***^ | 1.31^***^ | 1.41^***^ | 1.48^***^ |
|  | (3.40) | (3.89) | (4.74) | (4.14) | (4.01) | (5.21) |
| Years since junior prof² | 0.99 | 0.98^*^ | 0.97^**^ | 0.99^*^ | 0.98^*^ | 0.97^**^ |
|  | (-1.64) | (-2.04) | (-2.93) | (-1.96) | (-2.05) | (-3.15) |
| International publications (ln) | 1.04 | 0.97 | 0.97 | 1.18^*^ | 0.99 | 1.05 |
|  | (0.44) | (-0.32) | (-0.27) | (1.99) | (-0.15) | (0.59) |
| Months abroad (ln) | 1.04 | 1.04 | 1.09^+^ | 1.08^+^ | 1.06 | 1.08^+^ |
|  | (0.84) | (0.73) | (1.70) | (1.81) | (1.22) | (1.70) |
| Graduated abroad | 1.15 | 1.14 | 1.05 | 1.06 | 1.15 | 1.02 |
|  | (0.58) | (0.54) | (0.22) | (0.25) | (0.59) | (0.10) |
| PhD abroad | 1.82^*^ | 1.87^*^ | 1.39 | 0.97 | 1.71^*^ | 1.68^*^ |
|  | (2.41) | (2.52) | (1.43) | (-0.13) | (2.27) | (2.53) |
| Awards (ln) | 1.80^***^ | 1.82^***^ | 1.89^***^ | 2.17^***^ | 1.80^***^ | 1.86^***^ |
|  | (3.47) | (3.63) | (4.10) | (6.68) | (3.74) | (4.18) |
| DFG funding (ln) | 1.37^+^ | 1.34^+^ | 1.51^*^ | 1.13 | 1.56^**^ | 1.70^***^ |
|  | (1.89) | (1.77) | (2.46) | (0.96) | (2.70) | (3.56) |
| University of excellence | 0.61^**^ | 0.59^**^ | 0.53^***^ | 0.53^***^ |  | 0.57^***^ |
|  | (-2.82) | (-2.95) | (-3.44) | (-3.67) |  | (-3.38) |
| Studied: university of excellence |  |  |  |  | 1.10 |  |
|  |  |  |  |  | (0.54) |  |
| PhD: university of excellence |  |  |  |  | 0.83 |  |
|  |  |  |  |  | (-1.04) |  |
| Habil: university of excellence |  |  |  |  | 0.84 |  |
|  |  |  |  |  | (-0.94) |  |
| Mobility (ln) | 2.22^***^ | 2.24^***^ | 2.20^***^ | 2.22^***^ | 2.10^***^ | 2.40^***^ |
|  | (6.09) | (6.15) | (6.06) | (6.37) | (5.86) | (7.37) |
| Interim professor (ln) | 1.12 | 1.08 | 1.22 | 0.92 | 1.12 | 1.20 |
|  | (0.76) | (0.54) | (1.41) | (-0.72) | (0.84) | (1.41) |
| Co-authors (ln) | 1.05 | 1.08 | 1.07 | 1.14^+^ | 1.10 | 1.06 |
|  | (0.57) | (0.94) | (0.79) | (1.74) | (1.16) | (0.74) |
| Incomplete |  |  | 2.16^***^ | 1.75^*^ | 1.80^**^ | 2.32^***^ |
|  |  |  | (3.83) | (2.43) | (2.76) | (4.42) |
| Before 2002 | 1.19 | 1.16 |  | 0.60^***^ | 1.36^+^ | 1.37^*^ |
|  | (1.04) | (0.86) |  | (-3.39) | (1.82) | (2.10) |
| Nr children=1 |  |  |  |  |  | 1.19 |
|  |  |  |  |  |  | (0.74) |
| Nr children=2 |  |  |  |  |  | 1.69^*^ |
|  |  |  |  |  |  | (2.57) |
| Nr children=3 |  |  |  |  |  | 0.84 |
|  |  |  |  |  |  | (-0.48) |
| Nr children=4 |  |  |  |  |  | 0.22 |
|  |  |  |  |  |  | (-1.14) |
| Nr children=5 |  |  |  |  |  | 0.00 |
|  |  |  |  |  |  | (.) |
| Nr children=6 |  |  |  |  |  | 0.00 |
|  |  |  |  |  |  | (.) |
| Nr children=7 |  |  |  |  |  | 0.00 |
|  |  |  |  |  |  | (.) |
| Nr children=99 |  |  |  |  |  | 0.99 |
|  |  |  |  |  |  | (-0.06) |
| Female=1 |  |  |  |  |  | 1.16 |
|  |  |  |  |  |  | (0.55) |
| Nr children=1 # Female=1 |  |  |  |  |  | 1.04 |
|  |  |  |  |  |  | (0.11) |
| Nr children=2 # Female=1 |  |  |  |  |  | 0.44^+^ |
|  |  |  |  |  |  | (-1.86) |
| Nr children=3 # Female=1 |  |  |  |  |  | 4.06^*^ |
|  |  |  |  |  |  | (2.57) |
| Nr children=4 # Female=1 |  |  |  |  |  | 0.00 |
|  |  |  |  |  |  | (.) |
| Nr children=6 # Female=1 |  |  |  |  |  | 0.67 |
|  |  |  |  |  |  | (.) |
| Nr children=99 # Female=1 |  |  |  |  |  | 1.45 |
|  |  |  |  |  |  | (1.12) |
| r² | .13 | .14 | .14 | .094 | .13 | .14 |
| Individuals tenured | 299 | 299 | 288 | 356 | 313 | 356 |
| Individuals total | 1270 | 1270 | 1381 | 356 | 1308 | 1453 |
| Observations | 32726 | 32726 | 26308 | 14103 | 31342 | 35578 |

Notes: Exponentiated coefficients; t statistics in parentheses; cluster-robust standard errors;

^+^ *p* < 0.1, ^*^ *p* < 0.05, ^**^ *p* < 0.01, ^***^ *p* < 0.001

Table A5: Replication with exits to possibly permanent posts

|  | (1) | (2) | (3) | (4) | (5) | (6) | (7) |
| --- | --- | --- | --- | --- | --- | --- | --- |
|  | Gender | Publications | Signaling | Social capital | Children | Women | Men |
| Female | 0.91 | 1.21 | 1.36^*^ | 1.17 |  |  |  |
|  | (-0.76) | (1.58) | (2.29) | (1.11) |  |  |  |
| SSCI journal articles (ln) |  | 2.11^***^ | 1.71^***^ | 1.67^***^ | 1.66^***^ | 1.87^**^ | 1.61^***^ |
|  |  | (9.11) | (5.61) | (5.02) | (4.96) | (2.90) | (4.17) |
| Monographs, reputable (ln) |  | 1.53^**^ | 1.24 | 1.08 | 1.06 | 1.01 | 1.09 |
|  |  | (2.90) | (1.53) | (0.58) | (0.42) | (0.04) | (0.52) |
| Monographs, regular (ln) |  | 1.02 | 0.92 | 0.94 | 0.93 | 1.56 | 0.74^*^ |
|  |  | (0.12) | (-0.61) | (-0.48) | (-0.57) | (1.44) | (-2.02) |
| Non-SSCI journal articles (ln) |  | 1.18^+^ | 1.16 | 1.07 | 1.06 | 1.01 | 1.15 |
|  |  | (1.95) | (1.63) | (0.76) | (0.63) | (0.03) | (1.28) |
| Edited volumes (ln) |  | 1.34^*^ | 1.22 | 1.20 | 1.22 | 0.81 | 1.37^+^ |
|  |  | (2.41) | (1.57) | (1.39) | (1.47) | (-0.66) | (1.95) |
| Book chapters (ln) |  | 1.20^+^ | 1.34^**^ | 1.33^*^ | 1.33^*^ | 1.61^*^ | 1.26^+^ |
|  |  | (1.80) | (2.63) | (2.49) | (2.56) | (2.05) | (1.78) |
| Gray literature (ln) |  | 1.09 | 1.06 | 1.10 | 1.10 | 1.05 | 1.10 |
|  |  | (1.33) | (0.92) | (1.34) | (1.30) | (0.36) | (1.10) |
| Years since habilitation |  |  | 1.59^***^ | 1.50^***^ | 1.50^***^ | 1.58^**^ | 1.61^***^ |
|  |  |  | (7.02) | (5.65) | (5.79) | (3.13) | (6.03) |
| Years since habilitationÂ² |  |  | 0.97^***^ | 0.97^***^ | 0.97^***^ | 0.96^*^ | 0.97^***^ |
|  |  |  | (-4.12) | (-3.33) | (-3.43) | (-2.35) | (-3.45) |
| Years since ass prof |  |  | 1.48^***^ | 1.43^***^ | 1.45^***^ | 0.96 | 1.75^***^ |
|  |  |  | (5.52) | (4.63) | (4.98) | (-0.20) | (6.77) |
| Years since junior profÂ² |  |  | 0.98^**^ | 0.98^*^ | 0.98^**^ | 1.01 | 0.96^***^ |
|  |  |  | (-3.17) | (-2.55) | (-2.80) | (0.78) | (-4.08) |
| International publications (ln) |  |  | 1.00 | 1.01 | 1.01 | 0.97 | 1.03 |
|  |  |  | (-0.03) | (0.10) | (0.09) | (-0.15) | (0.26) |
| Months abroad (ln) |  |  | 1.09^*^ | 1.06 | 1.06 | 1.10 | 1.05 |
|  |  |  | (1.99) | (1.22) | (1.31) | (1.07) | (0.87) |
| Graduated abroad |  |  | 0.87 | 1.06 | 1.11 | 1.01 | 1.14 |
|  |  |  | (-0.67) | (0.29) | (0.49) | (0.02) | (0.55) |
| PhD abroad |  |  | 1.50^*^ | 1.73^*^ | 1.75^**^ | 1.18 | 2.47^***^ |
|  |  |  | (2.11) | (2.56) | (2.71) | (0.40) | (3.87) |
| University of excellence |  |  | 0.68^*^ | 0.61^**^ | 0.58^**^ | 0.58^+^ | 0.61^*^ |
|  |  |  | (-2.44) | (-3.03) | (-3.27) | (-1.85) | (-2.46) |
| Awards (ln) |  |  | 1.86^***^ | 1.74^***^ | 1.74^***^ | 2.29^**^ | 1.56^*^ |
|  |  |  | (4.10) | (3.61) | (3.66) | (3.04) | (2.32) |
| DFG funding (ln) |  |  | 1.85^***^ | 1.54^**^ | 1.63^**^ | 1.94^+^ | 1.67^***^ |
|  |  |  | (4.02) | (2.75) | (3.21) | (1.65) | (3.31) |
| Mobility (ln) |  |  |  | 2.30^***^ | 2.34^***^ | 2.07^**^ | 2.71^***^ |
|  |  |  |  | (6.93) | (7.11) | (3.15) | (6.66) |
| Interim professor (ln) |  |  |  | 1.12 | 1.12 | 1.30 | 1.04 |
|  |  |  |  | (0.84) | (0.88) | (1.06) | (0.23) |
| Co-authors (ln) |  |  |  | 1.06 | 1.07 | 0.98 | 1.09 |
|  |  |  |  | (0.69) | (0.79) | (-0.13) | (0.95) |
| childless # Female=0 |  |  |  |  | 1.00 |  | 1.00 |
|  |  |  |  |  | (.) |  | (.) |
| childless # Female=1 |  |  |  |  | 1.21 | 1.00 |  |
|  |  |  |  |  | (0.73) | (.) |  |
| with children # Female=0 |  |  |  |  | 1.42^+^ |  | 1.44^*^ |
|  |  |  |  |  | (1.85) |  | (1.99) |
| with children # Female=1 |  |  |  |  | 1.15 | 0.93 |  |
|  |  |  |  |  | (0.56) | (-0.24) |  |
| w/o child info # Female=0 |  |  |  |  | 1.01 |  | 1.00 |
|  |  |  |  |  | (0.07) |  | (-0.01) |
| w/o child info # Female=1 |  |  |  |  | 1.74^*^ | 1.18 |  |
|  |  |  |  |  | (2.41) | (0.53) |  |
| Before 2002 | 0.71^**^ | 0.82 | 1.15 | 1.19 | 1.23 | 1.08 | 1.37^+^ |
|  | (-2.58) | (-1.31) | (0.96) | (1.10) | (1.34) | (0.21) | (1.78) |
| Incomplete | 1.46^*^ | 2.31^***^ | 2.22^***^ | 2.28^***^ | 2.28^***^ | 2.43^*^ | 2.66^***^ |
|  | (2.35) | (4.06) | (3.98) | (4.15) | (4.27) | (2.21) | (4.34) |
| r2 | .0031 | .06 | .11 | .13 | .13 | .15 | .17 |
| Individuals tenured | 356 | 356 | 356 | 356 | 356 | 109 | 247 |
| Individuals total | 1450 | 1450 | 1450 | 1450 | 1450 | 549 | 901 |
| Observations | 33210 | 33210 | 33210 | 33210 | 33210 | 9685 | 23525 |

Notes: Exponentiated coefficients; t statistics in parentheses; cluster-robust standard errors;

^+^ *p* < 0.1, ^*^ *p* < 0.05, ^**^ *p* < 0.01, ^***^ *p* < 0.001
