# Supplementary material for: Publishing, signaling, social capital, and gender: Determinants of becoming a tenured professor in German political science
Source: PLoS One. 2021 Jan 6;16(1):e0243514. doi: 10.1371/journal.pone.0243514 (PMC7787375; doi:10.1371/journal.pone.0243514)
Supplement: Attachment — Submitted filename: PONEreview10-23-20202.docx [file pone.0243514.s002.docx]

Schröder, Lutter and Habicht have done an excellent job on an extremely important topic. One can quarrel with small aspects of their measures and analyses, but these are minor and can be addressed in a revision. One can also quarrel with their failure to cite relevant literature, but again this is a minor omission that can easily be rectified. To me, the importance of their study is that it avoids the common problem of non-representative sampling that limits much of the literature; moreover, it goes beyond the primary question, and they were able to rule out plausible alternative explanations related to productivity and gender bias. I wish these authors were somewhat bolder in their conclusions because they have some of the best evidence that argues against explanations involving bias, showing that if anything female political scientists are actually advantaged in the German system when controlling for various factors, most importantly productivity (i.e., at the professorship stage, women are appointed with fewer publications than men). As these authors point out, this is opposite to past claims of gender bias which argue that women need many more publications than men to achieve the same outcome (e.g., Wenneras & Wold reported this for getting postdoc fellowships in Sweden and many others have claimed it is true for professorial appointments). There are far better cites for this point than the two mentioned. And lest one argue that journals are biased against accepting submissions from female authors (Budden et al. 2008 made this claim, but it has been repeatedly refuted), the evidence when taken as a whole shows this is not true. For example, Berg (2017) reported similar acceptance rates for men and women at the journal *Science,* as have many others, with only a few exceptions.

There have been many studies of faculty hiring in the U.S. and readers will want to know how these findings compare with the present findings. Williams & Ceci (2015) can be contrasted with Moss-Racusin et al. (2012) and Eaton et al. (2019), although the former dealt with tenure-track hiring of faculty, whereas the second dealt with hiring of lab managers with only baccalaureate training, and Eaton et al. dealt with appointing of postdocs. They differed somewhat in fields (all three included biology but Eaton et al. and perhaps Moss-Racusin et al. may have included physics, and Williams & Ceci included psychology, engineering, and economics as well as biology). And an older study by Steinpreiss et al. (~late 90s) dealt with psychology only. Williams & Ceci dealt with outstanding finalists for tenure track assistant professorships, whereas Eaton et al. dealt with fairly weak postdocs and Moss-Racusin et al. dealt with BA-level lab manager applicants who were described as uneven (strong on some dimensions but unimpressive on others). None of these studies were full population analyses like Schröder, Lutter & Habicht’s. And none were able to rule out various hypotheses like theirs. Thus, the present study has some definite advantages and will be seen as a very important contribution to this literature, which by the way, is one of the most highly cited in all of social science. (One metric is citations: I seem to recall reading that Moss-Racusin et al., which did not deal with faculty hiring and tenure, has been cited around a thousand times as of a few months ago.)

There is also a literature on leakage from the pipeline that the authors might cite. And a large literature on gender gaps in productivity (note--NOT bias in article acceptance, but rather productivity itself, meaning how much work is produced). Basically, all studies find large gender gaps favoring males in amount of work published in journals (again, not bias in acceptance but rather differential output of work with men producing more), regardless of the order of author (first author, corresponding author, any order of authorship), field (some significant differences but all report male advantage), stage of career, nation, etc. Huang et al. (2020) analyzed the full publication history in the Web of Science for over 1.5 million authors and found a large gender gap in favor of men (27% more publishing over a lifetime), but there was no significant gender gap in median publications. Men work about 10% more years and have fewer career interruptions and are three times more likely to be at the extreme right tail of the productivity distribution (Kelchermans and Veugelers, 2013).

A large literature exists in America on the negative role of being a parent when applying for tenure track posts. In addition to the Mason et al. (2013) paper cited, she and Goulden and Frasch as well as Cech & Blair-Loy (2019) and many others have reported that in the U.S., women leave academic careers after childbirth about twice as often as men do following the birth of a child. Many surveys of postdocs and graduate students report that women are much more worried that employment in tenured posts may be incompatible with family life (e.g., Ecklund & Lincoln, 2011; Martinez, 2007). The presence of children or the plans to later have children both reduce women’s likelihood of applying for what Mason and her colleagues call “fast-track” academic careers, i.e., assistant professor posts at research intensive universities. Yet, as far as salary goes, women with young children are quite successful in the NSF Survey of Doctoral Recipients data base, earning higher salaries than those without children.

Minor:

The phrase “lower factual hiring rates of women” is misleading. Women are **not** actually hired at a lower rate than would be expected based on their representation in the applicant pool; they are underrepresented in the applicant pool, due to earlier leakage in social sciences (but not in physics, economics, CS, mathematics).
