# Supplementary material for: Publishing, signaling, social capital, and gender: Determinants of becoming a tenured professor in German political science
Source: PLoS One. 2021 Jan 6;16(1):e0243514. doi: 10.1371/journal.pone.0243514 (PMC7787375; doi:10.1371/journal.pone.0243514)
Supplement: Attachment — Submitted filename: Response to Reviewers_ML01.docx [file pone.0243514.s003.docx]

**Revision Memo for Submission PONE-D-20-23645**

Dear editorial team,

We would like to thank the two reviewers for the careful reading of our manuscript and for their highly constructive comments, which helped us to improve our study. We revised the paper carefully based on these suggestions. Please find our detailed response to each of the comments below.

| **No.** | **Reviewer Comments** | **Response to Reviewer** |  |
| --- | --- | --- | --- |
|  | **Editor comments** |  |  |
|  | Both reviewers appreciated the extent, depth and quality of the work, and made constructive minor comments that will improve its scholarly value and impact. As an editor I would add a minor comments about the frequent use of the term "insignificant" for statistical estimates. I would avoid this word, which as the authors surely know is inaccurate, particuarly when the entire population of interest is being measured (although only at a particular time, of course). I would encourage the authors to make greater use of e.g. "not statistically significant" adding where appropriate "at xxx level". Even better, perhaps the authors could consider running a post-hoc power analysis to determine what range of effect magnitudes would fail to reject the null, which would allow them to equate more clearly the failure to reject the null with a "small" (but not necessarily zero or "instignificant") effect. | We have removed every mention of the word “insignificant” and replaced it with “not statistically significant” everywhere. When an effect is statistically significant, we now usually mention at which level, but when it is not, we simply mention this as well. Some articles caution against the use of power analysis in observational data (see, for example, Hoenig / Heisey 2001: The Abuse of Power: The Pervasive Fallacy of Power Calculations for Data Analysis. In: The American Statistician, February 2001, Vol. 55, No. 1.) We therefore – as also suggested by the editor – report on significance levels and confidence intervals instead, which should be equivalent in making clear how much insignificant results miss the mark. |  |
|  |  |  |  |
|  | **Reviewer 1** |  |  |
| 1.1 | The authors have done an admirable job collecting and analyzing data on political science in Germany. Their analysis is the most extensive I have seen. And because they are essentially dealing with the entire population of German political scientists, one can have confidence that extraneous factors are not instrumental. I anticipate their findings will be highly cited; I know that my colleagues and I will cite it because it fills gaps that numerous published hiring audits cannot address. There are, however, several minor revisions that these authors could do to strengthen what is already a very strong paper. | Thank you for these comments, which we think we could all integrate. |  |
|  | First and foremost, they should situate their analysis within the broader international context where the issues of gender bias in academic hiring and promotion are most prevalent. In the present version of the manuscript very few of the major studies are acknowledged. For example, a number of empirical analyses of tenure-track hiring have been reported in the U.S. and Canadian literatures, mostly studies of hiring and promotion at U.S. universities. These studies have been of two types, analysis of gender gaps in actual hiring by universities (I will refer to these as audit studies) and quasi experiments in which faculty are asked to judge the same hypothetical CV for tenure-track hiring or promotion, with a male vs. female name on it. These studies have led to contradictory results. Steinpreiss et al. (1999) found that the hiring of new assistant professors in psychology favored applicants with a male name on the CVs as opposed to the same CV when it had a female name on it. (However, they reported that hiring for more advanced candidates for early tenure did not favor either gender.). Moss-Racusin et al. (2012) found that faculty favored hiring applicants whose CV had a male name over identical female applicants, for baccalaureate-level laboratory manager positions in biology. Easton et al. (2019) reported that faculty favored hiring male postdocs in physics but nonsignificant differences in hiring postdocs in biology.  Williams and Ceci (2015) found faculty in four fields favored hiring female applicants for assistant professor positions over male applicants with identical CVs, 66%-33%. In addition, there are numerous studies of the productivity of female applicants for research grants (e.g., Wenneras & Wold, 1997 reported that Swedish female postdocs in biomedicine had to have much greater productivity to get the same grant as a male postdoc received with far fewer publications; however, later, colleagues analyzed the same granting agency and did not find greater productivity by female applicants, in fact, they found that it favored males slightly, Sandstrom & Hallsten, 2007.). By the way, there is an enormous literature on sex differences in productivity, mostly documenting that men publish more at all author positions, in all cohorts, and in most disciplines (e.g., Xie & Shauman,1998; Huang et al. 2020--PNAS). In Huang et al.’s analysis of lifetime publications, political science had a large female advantage in annualized publications and a 3.3% female advantage in total impact. Some contact with this finding is called for as readers familiar with it will want to know why the authors think this occurred. Obviously, there are big differences in the sampling and analytic approach between these studies and the present German study. It would behoove the authors to acknowledge this larger literature and perhaps attempt to reconcile their contradictory findings. One promising avenue is the authors mention in several places the distinction between applying for jobs vs. being promoted after applying. In the American hiring audits there is a lot of evidence showing that women who earn PhDs are much less likely to apply for tenure-track jobs than are men who earn PhDs. | Thank you, we were only aware of some of these studies, have read them all and integrated each one. We therefore added Steinpreis et al. 1999, we also reviewed the results from Moss-Racusin et al. and mention that our results may be different because we look at a different discipline, which can imply that discrimination exists in the natural sciences, but not in (German) political science. We assume that with Easton et al. (2019) the reviewer meant Eaton et al. (2020) and have added it as well. We have also referred to Williams and Ceci 2015. However, we cannot resolve why some studies find discrimination of favor in women, while others find discrimination in favor of men. We can only say whose side of the argument our results tend to confirm. We have now cited Wenneras & Wold, 1997 as well as Sandstrom & Hallsten, 2007. Since we are not trying to explain who gets third party funding, we did not use them in our discussion, as our results do not refute or confirm them. But they were great in explaining why we need to control for the variables that we control for. Likewise, we used Xie and Shauman to argue why we have to control for career stages. Notably, their argument is that women publish less than men because they do not reach higher career stages. Similarly, we used Huang et al to argue why we have to account for publications. Huang et al show a productivity gap between men and women. They have therefore helped us to explain why we have to control for publications, namely to show whether the female productivity gap explains why women get hired less or whether women get hired less even if they have the same productivity. The reviewer is also suggesting that we look more specifically at the leaky pipeline phenomenon, according to which more women quit academia than men do. We now mention that the leaky pipeline phenomenon is why we control for career stages, allowing us to compare researchers who have made it to similar career stages in the first place. |  |
| 1.2 |  |  |  |
| 1.3 | The presence of children emerges in some past studies (the authors cite Mason et al. but there are many other studies that have found children do not pose a problem for women, once they are hired but their presence does deter women from applying in the first place. These authors’ fourth (or fifth, I forget) model rules this out nicely. And there are analyses of the effect of children as a function of academic discipline and rank (see Ceci, Ginther, Kahn & William’s analyses in the 2014 issue of Psychological Science in the Public Interest.) | We read through the very extensive review article by Ceci et al (44.000 words, more a book than an article) and contrasted our findings to their review. We also found that the Ginther / Shulamit piece on children, which was cited by Ceci et al., was useful to contrast our findings to. |  |
| 1.4 | Something should be said about the differences in cultural context that may be at work here. On the one hand, Germany has much more extensive child care during the early years than America and perhaps this could be at work. However, the presence of children and the linearity as a function of number of children suggest that childcare is not what is driving the differences between countries, with women who have three children being ~300% more likely to get tenure than childless women, a greater advantage than fathers over childless men. Goulden, Frasch, & Mason (2013, I think) reported that the presence of children or even plans for children in the future were associated with female attrition greater than male attrition among her sample of postdocs. While the authors touch upon gender differences in the willingness to apply for positions, perhaps they could say more as this seems critical to explaining the gender gap they observe. They make a huge contribution in showing that this is not the result of bias against women and that women publish less than men who are hired. Does German academic have something akin to affirmative action like in the U.S.? How else should one explain the seeming preferential tenuring of women with lower productivity? Anyway, there is an enormous literature—far too large to seriously review it—that the authors could selectively cite from to locate the present analysis in this broader context without claiming to have comprehensively reviewed it. However, even without this, the present study is very nicely done and I anticipate that it will be highly cited. | Similar to the reviewer and as mentioned in the article, we also believe that the effect is likely not due to children, because while women with two children have lower chances to get a professorship (however, the effect is not significant at the 5 % level), those with three children have a slightly higher chance. Our conclusion that children do not seem to make a difference is similar to the findings in the US that we cite. However, we now do mention in the conclusion that while children do not seem to stop women from getting a tenure track job when they apply, it is possible that they get women to leave academia altogether (we test this in a different article) and cite Goulden, Frasch, & Mason for his. We now also mention that one way to explain the preferential hiring of women is indeed affirmative action that favors women.  As far as we can tell, by incorporating the literature above, as well as the important points that have been raised, we have done what reviewer 1 suggested. Otherwise, please let us know what might still be missing and thank you for these comments. |  |
|  |  |  |  |
|  | **Reviewer 2** |  |  |
|  | Schröder, Lutter and Habicht have done an excellent job on an extremely important topic. One can quarrel with small aspects of their measures and analyses, but these are minor and can be addressed in a revision. One can also quarrel with their failure to cite relevant literature, but again this is a minor omission that can easily be rectified. To me, the importance of their study is that it avoids the common problem of non-representative sampling that limits much of the literature; moreover, it goes beyond the primary question, and they were able to rule out plausible alternative explanations related to productivity and gender bias. I wish these authors were somewhat bolder in their conclusions because they have some of the best evidence that argues against explanations involving bias, showing that if anything female political scientists are actually advantaged in the German system when controlling for various factors, most importantly productivity (i.e., at the professorship stage, women are appointed with fewer publications than men). As these authors point out, this is opposite to past claims of gender bias which argue that women need many more publications than men to achieve the same outcome (e.g., Wenneras & Wold reported this for getting postdoc fellowships in Sweden and many others have claimed it is true for professorial appointments). There are far better cites for this point than the two mentioned. And lest one argue that journals are biased against accepting submissions from female authors (Budden et al. 2008 made this claim, but it has been repeatedly refuted), the evidence when taken as a whole shows this is not true. For example, Berg (2017) reported similar acceptance rates for men and women at the journal Science, as have many others, with only a few exceptions. | Thank you for this positive evaluation of the article and these kinds words. We can understand that we should be a bit bolder in our conclusion, so we now mention in the discussion that ours is the first study to use a virtually complete sample of all German academic political scientists to show that women tend to be favored over men in the hiring process for tenured professorships, before and after controlling for various factors, most importantly productivity. This means that women get hired with fewer measurable publications than men do. We now also explicitly argue that this the opposite of what studies such as Wenneras & Wold and Moss-Racusin et al. show, who argue for bias against women when they apply. While Budden et al. (2008) argue that journals are biased against publishing women, Berg (2017) argues that this is not the case. However, for our purpose, it is not important whether journals do or do not advantage women, since we are only trying to explain who gets a professorship with a given number of publications, rather than wondering where these publications come from in the first place. |  |
| 2.1 | There have been many studies of faculty hiring in the U.S. and readers will want to know how these findings compare with the present findings. Williams & Ceci (2015) can be contrasted with Moss-Racusin et al. (2012) and Eaton et al. (2019), although the former dealt with tenure-track hiring of faculty, whereas the second dealt with hiring of lab managers with only baccalaureate training, and Eaton et al. dealt with appointing of postdocs. They differed somewhat in fields (all three included biology but Eaton et al. and perhaps Moss-Racusin et al. may have included physics, and Williams & Ceci included psychology, engineering, and economics as well as biology). And an older study by Steinpreiss et al. (~late 90s) dealt with psychology only. Williams & Ceci dealt with outstanding finalists for tenure track assistant professorships, whereas Eaton et al. dealt with fairly weak postdocs and Moss-Racusin et al. dealt with BA-level lab manager applicants who were described as uneven (strong on some dimensions but unimpressive on others). None of these studies were full population analyses like Schröder, Lutter & Habicht’s. And none were able to rule out various hypotheses like theirs. Thus, the present study has some definite advantages and will be seen as a very important contribution to this literature, which by the way, is one of the most highly cited in all of social science. (One metric is citations: I seem to recall reading that Moss-Racusin et al., which did not deal with faculty hiring and tenure, has been cited around a thousand times as of a few months ago.) | As suggested, we have now compared our findings both to Williams & Ceci (2015), as well as to Moss-Racusin et al. (2012), Eaton et al. (2020) and Steinpreis (1999). We also mention now how we improve over these studies by being able to analyze a full sample and by not looking at the perception of fictious candidates but rather at who actually got hired. We are of course happy to hear that the reviewer thinks our study has the same potential to be cited as Moss-Rakusin and others. |  |
| 2.2 | There is also a literature on leakage from the pipeline that the authors might cite. And a large literature on gender gaps in productivity (note--NOT bias in article acceptance, but rather productivity itself, meaning how much work is produced). Basically, all studies find large gender gaps favoring males in amount of work published in journals (again, not bias in acceptance but rather differential output of work with men producing more), regardless of the order of author (first author, corresponding author, any order of authorship), field (some significant differences but all report male advantage), stage of career, nation, etc. Huang et al. (2020) analyzed the full publication history in the Web of Science for over 1.5 million authors and found a large gender gap in favor of men (27% more publishing over a lifetime), but there was no significant gender gap in median publications. Men work about 10% more years and have fewer career interruptions and are three times more likely to be at the extreme right tail of the productivity distribution (Kelchermans and Veugelers, 2013). | Yes, we agree that this is a literature that is important to be cited and have therefore cited articles on the leaky pipeline, such as Sabatier / Musselin / Pigeyre (2015), Abels / Woods (2015), Long / Allison / McGinnis (1993) . We have now also cited Huang et al. (2020) as well as Kelchtermans / Veugelers (2013). |  |
| 2.3 | A large literature exists in America on the negative role of being a parent when applying for tenure track posts. In addition to the Mason et al. (2013) paper cited, she and Goulden and Frasch as well as Cech & Blair-Loy (2019) and many others have reported that in the U.S., women leave academic careers after childbirth about twice as often as men do following the birth of a child. Many surveys of postdocs and graduate students report that women are much more worried that employment in tenured posts may be incompatible with family life (e.g., Ecklund & Lincoln, 2011; Martinez, 2007). The presence of children or the plans to later have children both reduce women’s likelihood of applying for what Mason and her colleagues call “fast-track” academic careers, i.e., assistant professor posts at research intensive universities. Yet, as far as salary goes, women with young children are quite successful in the NSF Survey of Doctoral Recipients data base, earning higher salaries than those without children. | We now cite Goulden, Mason and Frasch (2011) as well as Cech & Blair-Loy (2019)  to indicate that indeed, women are more likely to leave academia than men after childbirth. We are now also citing Ecklund & Lincoln (2011) as well as Martinez et al. (2007) to argue that more women than men worry about combining an academic career and children. We will soon be preparing an article where we use a second wave of our data to see who we cannot find in academia any more, to then calculate why academics leave academia. In this case, we would use such articles for our main literature review. Here we have used them to motivate why we control for children. Please note also that we control for selective women departure from academia already. Because children may hinder women more than men to continue their career, we control for children themselves, but also for career stages. If childbirth gets women to drop out of academia before reaching a certain career stage, controlling for these career stages should account for this effect. |  |
| 2.4 | Minor:   The phrase “lower factual hiring rates of women” is misleading. Women are not actually hired at a lower rate than would be expected based on their representation in the applicant pool; they are underrepresented in the applicant pool, due to earlier leakage in social sciences (but not in physics, economics, CS, mathematics). | We have removed the phrase “lower factual hiring rates of women.”  We thereby hope that we were able to incorporate all comments to your satisfaction and thank you for these extremely helpful and detailed suggestions. |  |

Please submit your revised manuscript by Dec 12 2020 11:59PM. If you will need more time than this to complete your revisions, please reply to this message or contact the journal office at [plosone@plos.org](mailto:plosone@plos.org). When you're ready to submit your revision, log on to <https://www.editorialmanager.com/pone> and select the 'Submissions Needing Revision' folder to locate your manuscript file.

Please include the following items when submitting your revised manuscript:

 * A rebuttal letter that responds to each point raised by the academic
   editor and reviewer(s). You should upload this letter as a separate
   file labeled 'Response to Reviewers'.
 * A marked-up copy of your manuscript that highlights changes made to
   the original version. You should upload this as a separate file
   labeled 'Revised Manuscript with Track Changes'.
 * An unmarked version of your revised paper without tracked changes.
   You should upload this as a separate file labeled 'Manuscript'.
